# Carer Involvement in Liaison Emergency Assessments at Devon Partnership Trust: A Quality Improvement Project

**DOI:** 10.1192/bjo.2026.11478

**Published:** 2026-06-30

**Authors:** Tom Scurr, Katy Somerville, Merryn Anderson, Nikhal Giri, Thomas Manders

**Affiliations:** 1 University of Exeter, Exeter, United Kingdom; 2 Devon Partnership Trust, Exeter, United Kingdom

## Abstract

**Aims:**

1. To improve carer contact and involvement in Emergency Department (ED) assessments within Devon Partnership Trust (DPT) liaison teams, in line with Psychiatric Liaison Accreditation Network (PLAN) standards, NICE guidance on suicide and safety planning, and the Triangle of Care.

2. To increase carer contact during emergency assessments, and completion of the associated information-sharing form to 90% target.

**Methods:**

We began with a baseline audit of 60 emergency assessments in September 2024 across three DPT liaison teams (Torbay, Exeter, and North Devon), using carer-related standards from PLAN and NICE. This revealed carers were not routinely contacted, the information-sharing form infrequently completed, and existing guidance was not consistently followed.

A Quality Improvement project team, including a carer representative, was formed to address these issues–initially focusing on increasing routine carer contact and appropriate documentation. A qualitative survey of team members identified key barriers, which then formed a driver diagram and change ideas implemented from October 2025. Workplace culture change around carer contact emerged as a central priority. A monthly rolling audit of 5 patients per team was implemented to monitor progress.

**Results:**

Initial SPC chart data indicated changes remained within normal variability, particularly in documentation rates. As a result, we maintained focus on early project targets rather than progressing on to the next targets. Nonetheless, recent data shows a positive trend, with updated data to be presented in our poster.

**Conclusion:**

Carer involvement in ED assessments is essential for enhancing assessment quality and patient safety, and is supported by PLAN, NICE, and the Triangle of Care. However, achieving consistent change is complex, particularly in shifting workplace culture. Our ongoing strategies are beginning to show success, and the project will continue until September 2026.

